# New mechanistic insights of integrin β1 in breast cancer bone colonization

**DOI:** 10.18632/oncotarget.2788

**Published:** 2014-11-15

**Authors:** Laure Thibaudeau, Anna V. Taubenberger, Christina Theodoropoulos, Boris M. Holzapfel, Olivier Ramuz, Melanie Straub, Dietmar W. Hutmacher

**Affiliations:** ^1^ Institute of Health and Biomedical Innovation, Queensland University of Technology, Kelvin Grove, QLD, Brisbane, Australia; ^2^ TU Dresden, Biotechnology Center, Tatzberg, Dresden, Germany; ^3^ Orthopedic Center for Musculoskeletal Research, University of Wuerzburg, Brettreichstraße, Wuerzburg, Germany; ^4^ Department of Anatomical Pathology and Cytopathology, Pathology Queensland Central Laboratory, Royal Brisbane and Women's Hospital, Herston QLD, Australia; ^5^ Institute of Pathology, University Clinic Rechts der Isar, Technical University Munich, Trogerstr, Munich, Germany; ^6^ George W Woodruff School of Mechanical Engineering, Georgia Institute of Technology, Atlanta, GA, United States of America; ^7^ Institute for Advanced Study, Technical University Munich, Lichtenbergstraße, Garching, Germany

**Keywords:** bone colonization, breast cancer, β1 integrin, humanized bone models, tissue engineering

## Abstract

Bone metastasis is a frequent and life-threatening complication of breast cancer. The molecular mechanisms supporting the establishment of breast cancer cells in the skeleton are still not fully understood, which may be attributed to the lack of suitable models that interrogate interactions between human breast cancer cells and the bone microenvironment. Although it is well-known that integrins mediate adhesion of malignant cells to bone extracellular matrix, their role during bone colonization remains unclear. Here, the role of β1 integrins in bone colonization was investigated using tissue-engineered humanized *in vitro* and *in vivo* bone models. *In vitro*, bone-metastatic breast cancer cells with suppressed integrin β1 expression showed reduced attachment, spreading, and migration within human bone matrix compared to control cells. Cell proliferation *in vitro* was not affected by β1 integrin knockdown, yet tumor growth *in vivo* within humanized bone microenvironments was significantly inhibited upon β1 integrin suppression, as revealed by quantitative *in/ex vivo* fluorescence imaging and histological analysis. Tumor cells invaded bone marrow spaces in the humanized bone and formed osteolytic lesions; osteoclastic bone resorption was, however, not reduced by β1 integrin knockdown. Taken together, we demonstrate that β1 integrins have a pivotal role in bone colonization using unique tissue-engineered humanized bone models.

## INTRODUCTION

Breast cancer (BC) is the most commonly diagnosed cancer and the second cancer-related cause of death in women in the western world [1-3]. Due to improved screening methods and treatments strategies, the mortality rate from this disease has decreased significantly over the last decades [1-3]. However, BC patients who appear in complete clinical remission may already have dormant disseminated tumor cells present at secondary sites in the skeleton, lung, liver or brain, which can result in late recurrence and the development of metastases. The appearance of overt bone metastases marks the entrance into an incurable phase of the disease, as the currently available treatment options are rather palliative than providing a cure [4, 5]. Therefore, the mechanisms by which BC cells home to the skeleton and colonize the bone microenvironment need to be better understood.

During the process of dissemination from the primary tumor to bone, BC cells adhere to and communicate with the surrounding tissues. Once BC cells have reached the metastatic bone site, they interact with their new microenvironment, which comprises bone matrix and different bone-resident cells. Among other cell adhesion molecules, integrins are known to mediate cellular interactions between tumor cells and the bone matrix [6, 7]. Integrins play important roles in the development and progression of cancers, and several integrins have been shown to be overexpressed in different types of cancer [8, 9]. In the case of BC, integrins β1 and β3 expressed on tumor cells play major roles, not only during tumor development and invasion, but also during cancer cell homing and the establishment of metastatic lesions [10, 11]. For instance, β1 integrin expression in BC cells is necessary for the initiation and maintenance of tumor growth in mice [12-14] and promotes metastasis from the primary site [13, 15, 16]. It has been demonstrated that treatment with ATN-161, a fibronectin-derived peptide known to interfere with integrin α5β1 and αvβ3 binding, resulted in a marked decrease in the incidence and number of skeletal and soft tissue metastases after intracardiac injection of MDA-MB-231 BC cells in nude mice, suggesting also a possible role for these integrin heterodimers in BC osteotropism [17].

So far αvβ3 integrin has been the focus of most studies looking at factors involved in bone metastasis. For instance, various *in vivo* experiments have suggested that αvβ3 integrin increases the potential of human and murine BC cell lines to form bone metastases [18-20]. In addition, injection of murine mammary tumor cells (66cl4) that overexpressed integrin β3 into the tibia of syngeneic mice resulted in increased osteoclast recruitment and bone resorption compared to parental cells [19]. It was also shown that treatment with the integrin inhibitor cilengitide or a snake venom-derived disintegrin (trigramin), both targeting primarily integrin αvβ3, significantly reduced the volume of tumors in the bone and the extent of osteolytic lesions after BC cell injection into the hind leg of rodents [21, 22]. However, it still remains unclear whether αvβ3 integrins specifically influence bone colonization or rather the prior arrest of BC cell in the skeleton. In fact, mice treated with the selective αvβ3 inhibitor S247 presented a reduced incidence and size of osteolytic lesions only when it was administered prior to the intracardiac inoculation of MDA-MB-435 BC cells, while it did not have an effect on bone degradation after the tumor cells had already disseminated to the bone [23]. Similarly Zhao *et al*. observed that αvβ3-overexpressing MDA-MB-231 cells led to an increased skeletal tumor burden and bone destruction compared to control cells after intravenous inoculation, but not when the cells were injected directly into the tibial cavity [20]. Moreover, tumor cell proliferation in the bone microenvironment does not appear to be modulated by αvβ3 [19], thus suggesting that other factors might play an important role in the establishment of BC cells in bone.

Despite their known role in primary tumor progression and their identification as a prognostic marker of invasive BC [24, 25], to date very few studies have specifically investigated the role of β1 integrins in BC-induced bone colonization. In our previous work, we have used primary human osteoblastic cell-derived matrices (hOBM) as a model system to study human species-specific interactions occurring between bone-metastatic cancer cells and the bone matrix *in vitro* [26, 27]. We have thereby shown that β1 integrins mediate adhesion of metastatic BC cell lines to bone extracellular matrix (ECM) [27], as was also reported by others [28, 29]. In this work we hypothesized that humanized *in vitro* [26, 27] and *in vivo* models [30-32] will allow to dissect the role of β1 integrins during bone colonization by metastatic BC cells. In our previous work, we have shown that human tissue-engineered bone constructs (hTEBCs) recapitulate a physiological “organ” bone with human-derived components and serve as a metastatic site for human BC cells in a murine host [30]. In the presented work we show that β1 integrin knockdown reduces spreading, attachment, and migration of metastatic BC cells on hOBM *in vitro*. While no effect was seen on cell proliferation *in vitro*, a delayed onset and a significantly reduced rate of tumor growth was observed *in vivo* upon suppression of β1 integrin expression. Despite their role in modulating tumor cell proliferation in the bone, β1 integrins did not appear to influence osteoclast activation and bone resorption. Finally, using tissue-engineered bone microenvironments we demonstrate key roles of β1 integrins during bone colonization of BC cells, thus suggesting that β1 integrins are promising targets in the treatment of bone metastatic disease.

## RESULTS

### β1 integrins mediate BC cell interactions with hOBM

**Figure 1:**
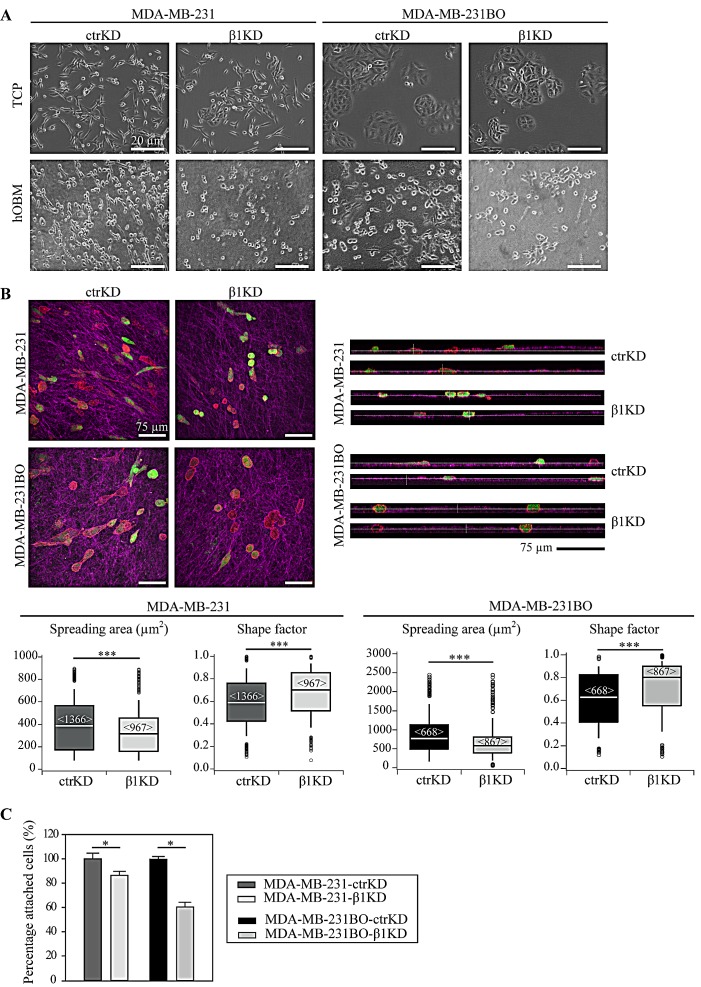
β1 integrins mediate BC cell spreading and attachment in a human-bone like microenvironment *in vitro* A: Representative phase contrast micrographs showing the morphology of ctrKD and β1KD BC cells grown on TCP and hOBM. B: Representative confocal z-stacks (maximal projections and orthogonal views) showing the morphology of GFP-positive (green) ctrKD and β1KD BC cells stained for F-actin (red) adhering for 24 hours on hOBM, which is visualized by an immunofluorescence staining against human-specific fibronectin (pink). Quantitative analysis of cell spreading area and shape factor. Box-plots show the medians, 75th and 25th percentiles; upper and lower whiskers indicate the 90th and 10th percentiles; circles denote outliers. Number of analyzed cells (from 6 different micrographs) is indicated in brackets. C: Quantification of cell attachment to hOBM. DNA content is measured after a 30 minutes attachment period and washing the cell layers. Data are represented as mean ± standard error.

In this study, we firstly investigated β1 integrin-dependent interactions of BC cells with human bone matrices *in vitro*, comparing sets of BC cells expressing endogenous β1 integrin and respective β1 knockdown cells. hOBM were derived from primary human osteoblastic cells (hOBs) as previously characterized [26, 27]. Two invasive BC cell lines, MDA-MB-231 and its bone-metastatic subline, MDA-MB-231BO [33], were chosen. MDA-MB-231BO cells were initially generated by Yoneda *et al*. through repeated *in vivo* passaging in mice by intracardiac injection and isolation from metastases in the skeleton [33]. Both cell lines were either transduced with control (ctrKD) or β1 integrin-targeting (β1KD) lentiviral shRNA vectors conjugated to green fluorescent protein (GFP). An efficient decrease in β1 mRNA and protein (cell surface and total) levels was confirmed using quantitative real-time polymerase chain reaction (qRT-PCR), flow cytometry, and western blots (Supplementary Figure 1). GFP signals were comparable between ctrKD and β1KD cells as demonstrated by flow cytometry. Firstly, the spreading morphology of individual ctrKD and β1KD BC cells on tissue-culture plastic (TCP) and hOBM was characterized (Figure 1A). Consistent with our previous work [27], we observed that all cell types underwent significant morphological changes when seeded onto hOBM; while more randomly oriented on TCP, they aligned their major axes parallel to each other on hOBM. Confocal laser scanning microscopy images revealed that BC cells aligned along the bone matrix fibers that were visualized using an immunofluorescent staining against human-specific fibronectin (Figure 1B). While the overall morphology of ctrKD and β1KD cells appeared similar on TCP (Figure 1A), quantitative image analysis demonstrated that β1KD cells were significantly less spread, and adopted a more rounded shape on hOBM compared to ctrKD cells (Figure 1B). Orthogonal views of the stained hOBM further indicated that a large proportion of both ctrKD and β1KD cells invaded and were partly embedded within the matrix (Figure 1B). To assess the effect of β1 integrins on cell attachment to hOBM, washing assays were performed. After an attachment period of 30 minutes, a higher percentage of ctrKD cells remained attached to hOBM after washing compared to β1KD cells (Figure 1C). Similar effects of β1 integrin knockdown on cell attachment were also observed using an additional BC cell line, SUM1315 (Supplementary Figure 2).

### β1 integrins enhance BC cell migration on hOBM but have no effect on BC cell proliferation *in vitro*

**Figure 2:**
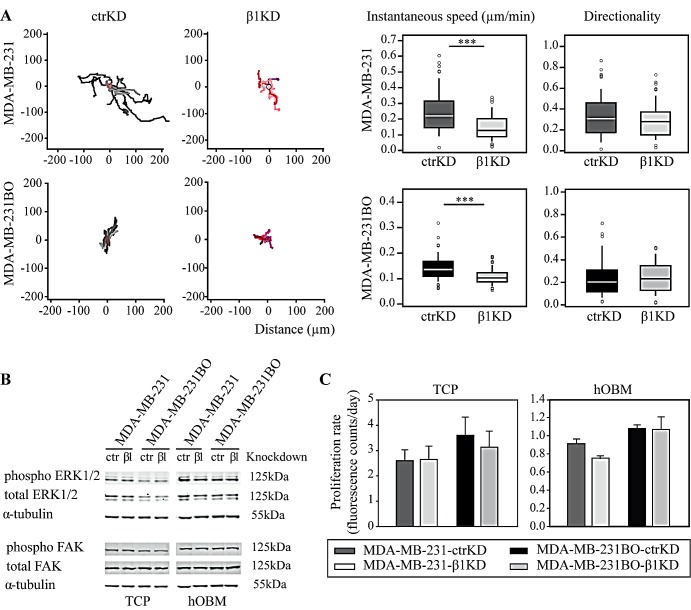
β1 integrins promote BC cell migration on hOBM but have no effect on ERK/FAK cell signaling and proliferation *in vitro* A: Representative tracks showing BC cell movement on hOBM and quantification of instantaneous migrational speed and directionality. Box-plots show the medians, 75th and 25th percentiles; upper and lower whiskers indicate the 90th and 10th percentiles; circles denote outliers. B: Western blot analysis of ERK1/2 and FAK phosphorylation in BC cells cultured on TCP and hOBM. C: Cell proliferation rates on TCP and hOBM evaluated by an Alamar Blue assay. Data are represented as mean ± standard error.

Next we assessed the effect of β1 integrin knockdown on BC cell migration on hOBM. For both cell lines, when compared to β1KD cells, ctrKD cells displayed an increased instantaneous migration speed on hOBM, while the directionality of cell movement was not affected (Figure 2A). In contrast, β1 integrin knockdown had no significant effect on BC cell migration when cells were seeded onto intact (i.e., non-decellularized) hOB cultures, which may be attributed to integrin-independent cell-cell interactions between BC cells and hOBs, or the reduced BC cell-bone ECM contacts in the presence of a dense cell layer (Supplementary Figure 3). Since focal adhesion kinase (FAK) and extracellular signal-regulated kinase (ERK) have been previously implicated in regulating cellular signaling pathways controlling cell migration, proliferation and survival [34-36], we analyzed phosphorylation of ERK1/2 and FAK using quantitative western blot analysis (Figure 2B, Supplementary Figure 4). In accordance with our previous study, ERK and FAK phosphorylation levels were increased on hOBM compared to TCP [27]. However, no changes in the phosphorylation levels of these signaling proteins were observed upon β1 integrin knockdown. In addition, similar proliferation rates were observed for ctrKD and β1KD cells grown on TCP or on hOBM (Figure 2C, Supplementary Figure 2). Similarly, ctrKD and β1KD cells grown in three-dimensional (3D) *in vitro* cultures, embedded into a gelatin-based hydrogel of a stiffness of 3.4 kPa, did not show any differences in cell proliferation rates nor in colony size of 3D spheroids (Supplementary Figure 5).

### β1 integrins promote tumor growth in the humanized bone microenvironment *in vivo*

**Figure 3:**
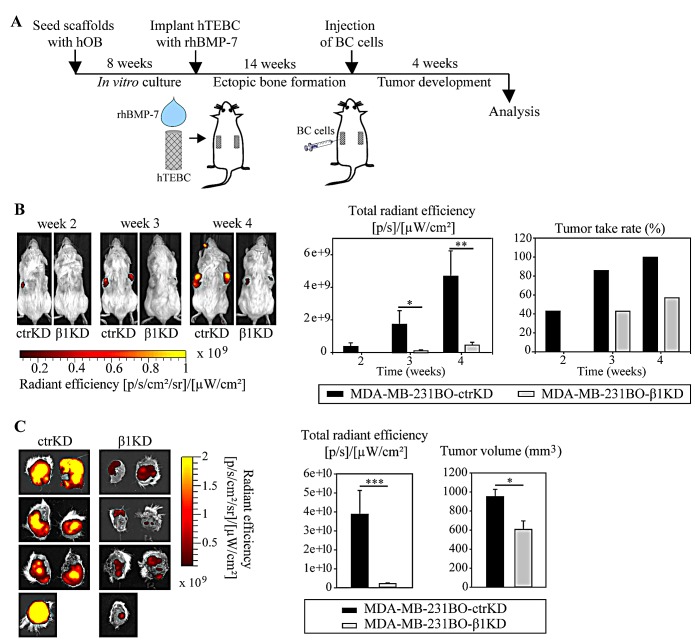
β1 integrins promote the development of larger GFP-expressing tumors in the bone microenvironment *in vivo* A: Schematic overview of the *in vivo* bone colonization experiment using the hTEBC model. B: Representative images and quantification of *in vivo* whole-body fluorescent imaging data over time. C: Images and quantification of *ex vivo* fluorescent imaging data at the experimental endpoint. Caliper measurements of tumor volume after excision. Data are represented as mean ± standard error.

We then sought to investigate the impact of β1 integrin knockdown on bone colonization using a humanized engineered bone model that has been recently established for the study of different steps of metastasis of osteotropic cancers [30-32]. Given their high predilection to form bone metastases in mice, MDA-MB-231BO ctrKD and β1KD were used. As shown earlier, MDA-MB-231BO cells are more successful in homing to and forming osteolytic lesions in the hTEBC than parental MDA-MB-231 cells [30]. To replicate a humanized bone microenvironment in mice, hOB-seeded scaffolds were implanted s.c. into the flanks of non-obese diabetic/severe combined immunodeficient (NOD/SCID) mice in combination with bone morphogenetic protein 7 (BMP-7). After 14 weeks, during which bone formation occurred and was verified by X-ray imaging (not shown), 1×10^6^ MDA-MB-231BO ctrKD or β1KD BC cells were injected into the center of the hTEBCs (Figure 3A). Development of GFP-positive tumors was monitored weekly by *in vivo* fluorescent imaging from week 2 onwards. A significantly increased fluorescence signal was detected for all analyzed time points for ctrKD cells compared to β1KD cells (Figure 3B). Furthermore, tumor growth was delayed until three weeks post-injection for β1KD injected constructs, while GFP signals of ctrKD tumors were firstly detected at week 2 (Figure 3B). At the 4-week endpoint of the experiment, tumors and scaffolds were excised, imaged *ex vivo*, and measured in size using calipers. GFP signals could be detected in all specimens from both ctrKD and β1KD groups *ex vivo*, which may be attributed to the attenuation of the fluorescence signal when imaging *in vivo* through the skin. However, consistent with the *in vivo* imaging data, significantly increased fluorescent signals together with larger tumors were observed in the control group compared to the group injected with β1KD cells (Figure 3C). qRT-PCR analysis revealed that β1 integrin levels in tumors formed in β1KD cell-injected scaffolds remained significantly decreased after 4 weeks *in vivo* (Supplementary Figure 6A), while GFP levels stayed constant in tumors from both groups, as confirmed by quantitative immunohistochemistry (IHC) analysis (Supplementary Figure 6B).

**Figure 4:**
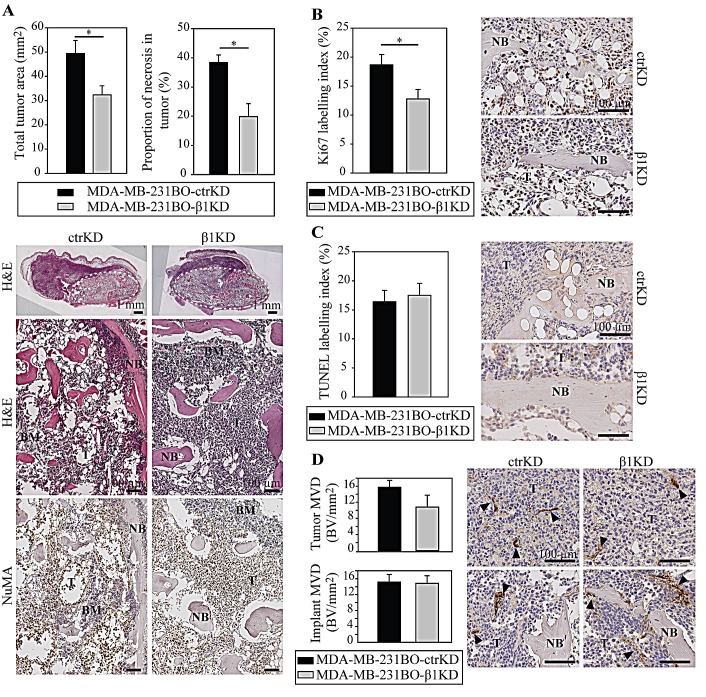
β1 integrins promote tumor growth in bone by increasing cell proliferation *in vivo* A: Analysis and representative images of H&E-stained tumor/hTEBC samples. Overviews and magnified images are shown (dotted line: boundaries of the hTEBCs). Detection of human-specific NuMA by IHC demonstrates the human origin of the cancer cells and osteoblasts. B: IHC detection of cell proliferation using the human-specific Ki67 marker. C: IHC analysis of cell apoptosis using a TUNEL stain. D: IHC analysis of vascularization in the implant and tumor using the vWF marker. Data are represented as mean ± standard error. BM: bone marrow, NB: new bone, T: tumor.

In line with the imaging results, histological analysis on hematoxylin and eosin (H&E)-stained sections from the tumor/hTEBC explants showed that tumor areas were larger in the ctrKD group (Figure 4A). Necrosis was seen mainly in the center of the larger tumors and, consistently with increased tumor sizes, increased necrotic areas were also observed in the ctrKD group. The tumors from both cell groups occupied most of the inside, and also grew outside of the engineered bone. The human origin of the tumor cells was demonstrated using IHC staining with a human nuclear mitotic apparatus protein (NuMA)-specific antibody (Figure 4A). Using IHC analysis of cell proliferation based on the Ki67 marker, it was demonstrated that BC cells with β1 integrin knockdown proliferated significantly less compared to controls (Figure 4B, Supplementary Figure 7). However no significant differences in tumor cell apoptosis and tumor vascularization could be detected (Figure 4C,D).

### β1 integrins do not modulate BC-induced bone resorption

**Figure 5:**
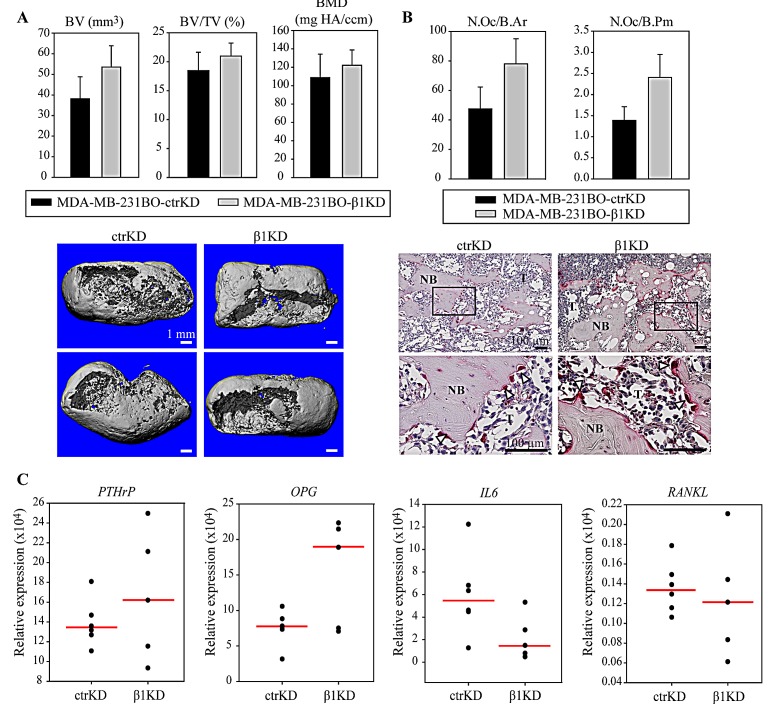
β1 integrins do not influence BC-induced bone resorption and osteoclast activation *in vivo* A: Representative 3D reconstructions of μ-CT data and quantification of BV, BV/TV and BMD. B: Histomorphometric analysis of osteoclast density normalized to the mineralized tissue area (N.Oc/B.Ar) or perimeter (N.Oc/B.Pm) and representative images of TRAP staining at low and high magnification. C: qRT-PCR analysis of PTHrP, OPG, IL-6 and RANKL gene expression in tumors formed in hTEBCs. Data in bar charts are represented as mean ± standard error. Dot plots represent individual data points and median. NB: new bone, T: tumor.

We next assessed whether knockdown of β1 integrins influenced BC-induced osteolysis. *Ex vivo* micro-computed tomography (μ-CT) analysis of the average mineralized tissue volume (BV), mineralized tissue volume fraction (BV/TV) and bone mineral density (BMD) were performed, but the differences were not statistically significant between groups (Figure 5A). Next, histomorphometric analyses were performed on histological sections of the samples but, consistent with the μ-CT results, no differences in the amount of bone could be detected between the ctrKD and β1KD groups (Supplementary Figure 8). The bone in contact with the cancer cells had obvious osteolytic changes in the presence of tumors from both cell groups and the presence of osteoclasts in resorption pits along the bone surfaces was confirmed by tartrate-resistant acid phosphatase (TRAP) staining (Figure 5B). Cells positive for TRAP - presumably macrophages - also were observed in the tumor not adjacent to bone (Supplementary Figure 8). Histomorphometric analyses were performed to quantify the number of multinucleated and TRAP-positive osteoclasts on the bone surface; however no significant differences in the average number of osteoclasts normalized to the mineralized tissue area (N.Oc/B.Ar) and perimeter (N.Oc/B.Pm) were observed in the presence of tumor cells from the ctrKD or β1KD group (Figure 5B). This is consistent with qRT-PCR results, which did not detect a significant difference in the gene expression of the osteoclastic factors parathyroid hormone-related protein (PTHrP), osteoprotegerin (OPG), interleukin 6 (IL-6) and receptor activator of nuclear factor kappa-B ligand (RANKL) when β1 integrins were decreased (Figure 5C).

### Tumor-harboring hTEBCs mimic closely clinical bone metastases from BC patients

**Figure 6:**
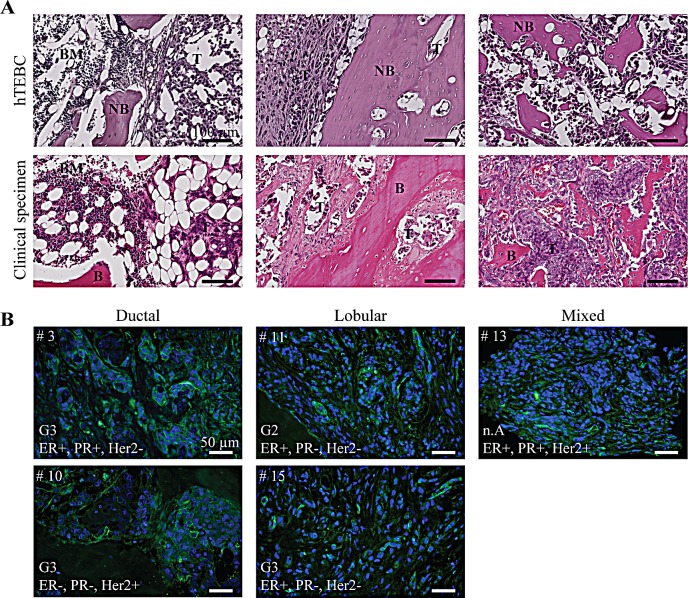
Tumor development in the hTEBC is representative of clinical bone metastases from breast carcinoma patients A: Representative H&E images showing the similarity between the tumor-harboring hTEBCs and clinical specimens at the histological level. B: IHC stainings of β1 integrin expression in bone metastases from patients with ductal, lobular or mixed mammary carcinoma. β1 integrins are labelled with Alexa Fluor 488 dye (green) and nuclei counterstained with DAPI (blue). Patient case number (#), histopathological grading score (G), and hormone receptor status (ER/PR/HER2: estrogen/progesterone/human epidermal growth factor receptor) for the bone metastases are shown. BM: bone marrow, NB: new bone, B: bone, T: tumor.

In order to correlate the observations made in our *in vivo* model with the clinic, we next compared histological sections of tumor-injected hTEBCs and clinical specimens of bone metastases. The latter consisted of tissue micro-arrays assembled from bone metastases from a cohort of 22 patients with breast carcinoma. H&E stainings revealed that bone metastases generated in the engineered bone bore a great resemblance to the clinical specimens (Figure 6A). In both cases, the cancer cells invaded the hematopoietic niche and were intermingled with other cell types such as adipocytes or hematopoietic cells (Figure 6A, left panel). The tumors invaded all the inter-trabecular spaces, gradually replacing the bone marrow and inducing the break-down of the bone matrix (Figure 6A, middle and right panels).

Our *in vivo* experiments are based on the use of the triple negative MDA-MB-231BO cell line which expresses high levels of β1 integrins compared to other BC cell lines [27], whereas patient tumors may exhibit varied molecular profiles depending on their subtype or histopathological grading. Thus, we next analyzed β1 integrin expression in the clinical tissue specimens. β1 integrin expression was detected in most cases using IHC, although staining intensity varied significantly across patient samples, with 5 cases showing moderate-to-strong immunoreactivity while other cases had a more diffuse cytoplasmic positive staining. Although variable β1 integrin expression levels were observed in the clinical specimens, β1 integrins could be detected in bone metastases of different subtypes of breast carcinoma and with different grading and hormone receptor status (Figure 6B).

## DISCUSSION

The critical roles of integrins in BC metastasis to bone have been widely described in the literature [10, 11], and these cell adhesion receptors are considered a promising target for developing new treatment strategies. In particular, β1 and β3 integrins have both been shown to promote osteotropic metastasis; however, whether they predominantly mediate tumor cell dissemination to, or colonization of the distant bone site by BC cells is not fully understood. To date, only αvβ3 integrins have been specifically studied in the context of BC-induced bone colonization. In this study, we investigated the specific role of β1 integrins in bone colonization by metastatic BC cells using our recently developed *in vitro* and *in vivo* humanized bone models.

We firstly observed that BC cells with reduced β1 expression adopted a more rounded morphology on the hOBM. This has been observed previously for mouse mammary cancer cells expressing shRNA targeting β1 integrin expression when they were cultured on basement membrane extract and collagen type I [37]. Additionally, in this study, β1KD cells adhered significantly less to bone ECM (hOBM) than controls. This is in accordance with our previous results which showed a decrease of BC cell adhesion to hOBM in the presence of a β1 integrin function-blocking antibody [27]. Similarly, it has been previously demonstrated that β1, α1, α2, and α3 integrin neutralizing antibodies strongly inhibited adhesion of BC cells to type I collagen or bovine cortical bone chips [28, 29]. In another study, pre-treatment of BC cells with snake venom disintegrins, such as rhodostomin, trigramin, and triflavin, reported to interfere with the function of αIIbβ3, α5β1 and αvβ3 integrins, inhibited adhesion of tumor cells to unmineralized and mineralized cell-secreted matrices produced by either MG-63 cells osteosarcoma cells or differentiated osteoblasts [22]. Furthermore, it has been shown that αvβ3 overexpression does not increase tumor cell adhesion to bone matrix proteins [19] and that blocking αvβ3 does not affect cells attachment to hOBM or bovine bone chips [27, 28]. Overall, these findings indicate that β1 integrins mediate BC cell adhesion to bone matrix proteins, and there are clues that β3 integrins may only play a minor role.

Using transwell assays, it has been shown before that disintegrins with selectivity for both β1 [22, 38] and β3 integrins [21, 22] inhibit chemotactic migration of BC cells toward ECM proteins, conditioned media or serum. In this study, we further observed that perturbed β1 integrin expression reduced the ability of BC cells to migrate along the hOB-secreted ECM fibers in the hOBM. This is consistent with previous reports showing β1 integrin-dependent directional migration of BC cells within 3D fibroblast-secreted ECM matrices [39], which was mainly attributed to β1 integrin binding to fibronectin fibers that are abundantly present in fibroblast-, but also our hOB-secreted matrices. We subsequently investigated the effect of β1 knockdown on cell proliferation and the regulation of ERK1/2 and FAK signaling pathways. β1 integrins did not influence BC cell proliferation in either TCP, hOBM or 3D hydrogels. Moreover, no effect on ERK and FAK phosphorylation levels was observed. Similar results have been obtained before on two-dimensional surfaces after knockdown of β1 integrins [40]. However, our results in 3D hydrogels are in contrast to previous studies, which demonstrated a significant decrease in proliferation in 3D ECM gels after disruption of the β1 integrin-FAK signaling axis in tumor cells [14, 40]. This discrepancy could be explained by the use of laminin-rich hydrogels to culture the BC cells, as opposed to the gelatin-based hydrogels used in our work. Since integrin downstream signaling pathways are also regulated by other integrin subunits and receptors, it is conceivable that the effect of β1 knockdown on downstream targets is highly substrate and cell type specific.

In order to investigate the role of β1 integrins on bone colonization *in vivo*, we next assessed tumor development after injection of metastatic BC cells into an ectopic humanized bone ossicle. In contrast to the *in vitro* result, β1 knockdown had a significant effect on tumor development in bone *in vivo*. FAK is known to integrate integrin and growth factor signaling [36], which may explain the different effects of β1 knockdown *in vitro* and *in vivo*, where the activation of both signaling axes is expected to be very different from each other. The reduction of β1 integrin expression in BC cells resulted in a delayed and reduced tumor growth in the hTEBCs, although tumor development was not prevented. Analysis by quantitative IHC revealed that the differences in tumor size within the humanized bone ossicle were related to a decreased proliferative potential in β1KD cells compared to ctrKD, while no differences in cell apoptosis and tumor vascularization were observed. This result is consistent with a recent study, which showed that loss of β1 integrins in PyVmT-induced mammary tumors in transgenic mice inhibited the proliferative capacity of tumor cells, but did not contribute to apoptotic cell death [12, 13]. Moreover the use of a specific inhibitory antibody against β1 integrins (AIIB2) also resulted in decreased mammary tumor formation [14]. Next to a decreased proliferative potential, another mechanism likely to play a role in the reduced tumor growth observed in the β1KD group is the impaired initial adhesion of the tumor cells to the ECM of the humanized bone. Although cell attachment to the bone matrix could not be quantified *in vivo*, this hypothesis is consistent with our *in vitro* results and with the observed initial delay in tumor development after injection, which suggests that β1 integrins may play a predominant role early in the bone colonization process. Only few studies so far have investigated the role of integrins during BC cell proliferation in the bone microenvironment *in vivo*. MDA-MB-231 BC cell proliferation in the tibia of nude mice was markedly inhibited using trigramin, a snake venom-derived disintegrin which binds to αIIβ3, αvβ3 and α5β1 [22]. Similar to the studies with integrin-blocking antibodies, this approach does not target a specific integrin subunit and can influence other integrin β1 and β3-expressing cell types such as endothelial cells or osteoclasts. However, it has been shown that αvβ3 overexpression does not affect 66cl4 cell proliferation in mouse tibia [19], thus indicating that this family of adhesion receptors is not critical for BC cell proliferation in bone. Thus, to our knowledge, we show here for the first time that β1 integrins expressed on BC cells play a significant role in the regulation of tumor growth in bone *in vivo*.

Despite decreased tumor size in animals injected with β1KD cells, we did not observe any significant differences in bone resorption and osteoclast activation between groups. This result indicates that β1 integrins do not play a major role in the induction of osteolytic lesion by metastatic BC cells. It also underlines the fact that an increased ability of BC cells to promote tumor expansion in the bone microenvironment does not necessarily correlate with an increased ability to recruit osteoclasts and promote bone resorption, as it has been shown before [19]. Since other studies have seen effects of αvβ3 integrin inhibitors on osteolytic lesions [21, 22], this may suggest that possibly αvβ3 integrins may be more important in osteolysis. However, the employed integrin inhibitors act not only on BC cells, but also on osteoclasts. Thus the effect of αvβ3 inhibitors on osteolytic activity may be predominantly due to the inhibition of β3 integrins on osteoclasts, which are critical in osteoclast-mediated bone resorption [41]. For instance, the αvβ3 inhibitor S247 was shown to induce significant morphological changes and impair formation of the actin sealing zone in osteoclast cultures [23].

In conclusion, using engineered human-bone mimicking *in vitro* and *in vivo* models we were able to delineate specific roles for tumor β1 integrins in the colonization of bone by metastatic BC cells. We have shown that β1 integrins promote the adhesion and migration of BC cells within bone matrix *in vitro* and promote tumor growth in bone *in vivo*. Using IHC analysis on a tissue micro-array assembled from a cohort of 22 patients we have shown that β1 integrins are detected in clinical bone metastatic tissue across different BC subtypes, although expression levels vary considerably between specimens. To date, three β1 integrin inhibitors (ATN-161, volociximab, and JSM6427) have been tested in clinical trials and show some promising results in the inhibition of tumor growth and distant metastasis in patients with solid tumors [42]. The new mechanistic insights of our study underline the potential of β1 integrins as a therapeutic target to limit tumor initiation and expansion in patients with bone metastases. Since not only BC growth, but also an inhibition of the bone-destructive action induced by BC cells is of great therapeutic relevance, the use of combination therapies with other inhibitors (i.e. against β3 integrins) may be necessary to efficiently target BC bone colonization and ultimately improve patient outcomes.

## MATERIALS AND METHODS

### Cell lines

Bone-metastatic BC cell lines SUM1315, MDA-MB-231, and MDA-MB-231BO were utilized in this study. MDA-MB-231 cells were purchased from the American Type Culture Collection (Manassas, Virginia, USA). MDA-MB-231BO cells were kindly provided by the University of Texas Health Science Center at San Antonio (San Antonio, Texas, USA) and SUM1315 cells were a gift from David Kaplan from the Tufts University (Medford, Massachusetts, USA). MDA-MB-231 and MDA-MB-231BO cells were maintained in high glucose Dulbecco's Modified Eagle Medium supplemented with 10% fetal bovine serum (FBS), 100 U/mL penicillin, 100 μg/mL streptomycin and 1x Glutamax, all sourced from Life Technologies (Mulgrave, Victoria, Australia). SUM1315 cells were cultured in F12 medium (Life Technologies) supplemented with 5 μg/mL human insulin (Sigma-Aldrich, St. Louis, Missouri, USA), 10 ng/mL epidermal growth factor (Sigma-Aldrich), 10% FBS and penicillin/streptomycin. BC cell lines were modified by retroviral transduction to stably express shRNA targeting integrin β1 RNA (β1KD), while control cells (ctrKD) expressed shRNA specific for firefly luciferase-GL2 RNA, together with a GFP reporter. The virus particles were kindly provided by T. Kwok [43]. Knockdown of integrin β1 was confirmed by qRT-PCR, flow cytometry, and immunoblotting (Supplementary Figure 1), as previously described [27].

### Preparation of hOBM

hOBs were isolated from bone tissue obtained under informed consent from female patients undergoing hip or knee replacement surgery (ethics approval number 0600000232), as described previously [26, 27]. hOBM for subsequent *in vitro* assays were prepared as previously described [26, 27]. Briefly, hOBs were seeded at a density of 3000 cells/cm^2^ onto thermanox^TM^ coverslips (Nunc, Thermo Fisher). Upon reaching confluency, cells were cultured under osteogenic conditions, in cell culture medium supplemented with 50 μg/ml ascorbate-2-phosphate, 10 mM β-glycerophosphate, and 0.1 μM dexamethasone (Sigma-Aldrich, Australia). After 4 weeks, mineralized matrices were decellularized using 20 mM ammonium hydroxide following a previously described protocol [26, 27].

### *In vivo* hTEBC model

All procedures were approved by the Queensland University of Technology Animal Ethics Committee (ethics approval number 0900000915) and carried out in accordance with the *Australian Code of Practice for the Care and Use of Animals for Scientific Purposes*. Four-week old female NOD/SCID mice were purchased from the Animal Resources Centre (Canning Vale, Western Australia, Australia) and maintained under specific pathogen-free, temperature-controlled conditions at the Pharmacy Australia Centre of Excellence animal facility (University of Queensland, Queensland, Australia). Animal experiments were performed using our previously described hTEBC model [30-32]. Briefly, calcium phosphate-coated melt electrospun polycaprolactone scaffolds were seeded with hOBs and cultured in osteogenic differentiation media for 8 weeks. Constructs were implanted s.c. in the flanks of the animals in combination with recombinant human BMP-7 (Olympus Biotech Corporation, Hopkinton, MA) and fibrin glue (TISSEEL Fibrin Sealant, Baxter Healthcare International, Deerfield, IL). New bone was allowed to form for 14 weeks and monitored at 4-6 weeks intervals with X-ray radiography. One million BC cells were then injected transcutaneously into each construct (n=7 hTEBCs per group) and tumor development was monitored weekly by *in vivo* fluorescent imaging using a Xenogen IVIS Spectrum (PerkinElmer, Waltham, Massachusetts, USA). At the experimental endpoint, hTEBCs were excised and analyzed by *ex vivo* fluorescent imaging. Image acquisition and analysis was performed using the spectral unmixing mode in the Living Image software (PerkinElmer). Signals were quantified by drawing an automatic region of interest with a threshold set at 10% around each fluorescent signal. Only signals above 800 counts were considered positive, a value which is more conservative than the threshold recommended by the manufacturer. Gross tumor measurements were performed with calipers upon explantation. Then, tumor specimens were either snap frozen in liquid nitrogen and stored at −80C before RNA extraction, or fixed in 4% paraformaldehyde overnight and then transferred to 70% ethanol for further analysis. In addition, *ex vivo* μ-CT analysis was performed on fixed samples as previously described [30].

### Histology and immunohistochemistry

Fixed samples were decalcified for 5 weeks in 10% ethylenediaminetetraacetic acid (pH 7.4) with weekly changes, and subsequently embedded in paraffin. Samples were stained routinely with H&E for morphological analysis of the tissue. To detect specific target proteins of interest, IHC was performed following a previously described protocol [30]. Primary antibodies used for IHC analysis are listed in Supplementary Table 1. Terminal deoxynucleotidyl transferase dUTP nick end labeling (TUNEL) staining was performed to detect apoptotic cells using a DeadEnd™ Colorimetric TUNEL System (Promega, Madison, WI, USA) according to the manufacturer's instructions. TRAP staining to detect osteoclasts was performed as previously described [30]. Sections were scanned at X20 magnification using a Leica SCN400 slide scanner (Leica Microsystems, Wetzlar, Germany) before image analysis.

### Image analysis

To quantify histochemical and IHC stainings, at least five different samples (hTEBCs) were assessed per experimental group, with two sections each (one from the central region of the sample; one approximately separated by 150 μm from the first section). The open source web-based ImmunoRatio application (Institute of Medical Technology, University of Tampere, Tampere, Finland) was used for automated image analysis of Ki67, GFP and TUNEL expression [44]. ImmunoRatio calculates a labeling index which corresponds to the percentage of diaminobenzidine-stained area over total hematoxylin-stained area. To validate this method, ImmunoRatio analysis of the Ki67-stained sections was compared to the analysis using ImageJ (Supplementary Figure 7). Quantification of microvessel density (MVD) was performed by manual counting of von Willebrand factor (vWF)-positive blood vessels in the entire sections and numbers were normalized to the tissue area (“implant” and “tumor” outside implant were quantified separately). Histomorphometry analysis on TRAP-stained sections was performed using the Osteomeasure software (OsteoMetrics Inc., Atlanta, GA, USA) to quantify the number of osteoclasts per bone area and perimeter. Only multinucleated, TRAP-positive cells on the bone surface were considered osteoclasts. TRAP-positive cells in the tumor area were also counted separately and normalized to total tumor area.

### Statistical analysis

Datasets were analyzed using the SigmaPlot software (Systat Software Inc, San Jose, California, USA). Normally distributed data was analyzed for statistical differences between groups using a Student t-test (2 groups) or a one-way ANOVA (> 2 groups). Data that failed the normality test was analyzed either using a Mann-Whitney test (2 groups) or a Kruskal-Wallis one-way ANOVA on ranks (> 2 groups). P < 0.05 was considered significant.

## SUPPLEMENTARY MATERIAL FIGURES AND TABLES


