# Supplementary material for: New mechanistic insights of integrin β1 in breast cancer bone colonization
Source: Oncotarget. 2014 Nov 15;6(1):332–44. doi: 10.18632/oncotarget.2788 (PMC4381598; doi:10.18632/oncotarget.2788)
Supplement: Supplementary file 1 [file oncotarget-06-332-s001.pdf]

New mechanistic insights of integrin β1 in breast cancer bone colonization

Supplementary Material

Flow cytometry

Cells growing at approximately 75% confluence were harvested using TripLE (Invitrogen) and resuspended at 10^6 cells/ml in ice-cold buffer A (=PBS containing 0.9 mM $CaCl_2$, 0.5 mM $MgCl_2$, 2% BSA, and 0.02% sodium-azide). After a 15 minutes blocking step on ice, 100 µl cell suspension (10^5 cells) were incubated in duplicate with mouse anti human antibodies specific to β1 integrins (P4G11, DSHB, 4 µg/ml,), αvβ3 integrins (LM609, Milipore, 10µg/ml) or isotype control antibodies (Invitrogen) for 1 hour on ice. Subsequently cells were washed with 1.5 ml buffer A and resuspended in 100 µl buffer A containing 5 µg/ml anti-mouse Alexa 633- conjugated secondary antibodies (Invitrogen). After 45 minutes incubation on ice in the dark, cells were washed twice in PBS containing 0.9 mM $CaCl_2$ and 0.5 mM $MgCl_2$ and fixed for 20 minutes in PBS containing 1% formalin, 2 % glucose, 0.01% sodium azide (RT). Data collection was performed on a Cytomics FC500 (Beckman Coulter Cytomics) flow cytometer. Acquired data were analyzed using the FlowJo software (Treestar Inc.). Mean fluorescence intensities were used for quantification. Additionally, histograms showing the distribution of fluorescence intensities were generated.

Western blot analysis

BC cells were seeded for 1.5 hours onto TCP or hOBM and then lysed in 200 µl sample buffer (62.5 mM Tris-HCl (pH 6.8), 2% (w/v) sodium dodecyl sulphate, 10% glycerol, 50 mM dithiothreitol, 0.01% (w/v) bromophenol blue). After 5 minutes at 95°C, 20 µl lysates were loaded onto 15% polyacrylamide gel and electrophoretically separated (120 V, 2 hours) in running buffer (24 mM Tris base, 20 mM Glycine, and 3.5 mM SDS). Then proteins were transferred onto a nitrocellulose membrane overnight (40 V, 4°C) (transfer buffer 25 mM Tris base, 20 mM glycine, 0.7 mM SDS, 20% methanol). Blots were blocked with 5% BSA/TBST

(20 mM Tris base, 140 mM NaCl, 0.1% Tween-20) or 5% low fat milk powder/TBST blocking buffer before incubation with primary antibodies (phospho-FAK tyr397 (clone 18), total-FAK (clone 4.47) (both Millipore), β1-integrin, phosphoERK1/2, total ERK1/2 (Cell Signaling) overnight at 4°C. Alpha-tubulin (clone 12G10, DSHB) was used as loading control. After washing in TBS-T, blots were incubated with IRdye680 and IRdye800 conjugated secondary antibodies (0.5 µg/ml, LICOR) for 2hours. Fluorescent bands were visualized using the Odyssey scanner (LICOR). Bands were analyzed using Image J (NIH).

Cell morphology and attachment assays

BC cells were seeded at a density of 2500 cells/cm^2 (for morphology assessment) or 10000 cells/cm^2 (for attachment assessment) on TCP or hOBM. Cell spreading area and shape factor were analyzed after 24 hours of culture. The shape factor is a quantity used to describe the circularity of the cells and is calculated as $4\pi[area]/[perimeter]^2$. A value of 1 indicates a perfect circle and a value approaching 0 indicates an increasingly elongated shape. Cells were stained by immunofluorescence with 10 µg/ml human fibronectin-specific antibody (HFN 7.1, DSHB), followed by 10 µg/ml secondary fluorescently labeled antibody (anti-mouse Alexa 633, Cell signaling), 0.8 U/ml rhodamine-conjugated phalloidin and 5 µg/ml 4',6-diamidino-2-phenylindole (DAPI) and imaged using a Leica TCS SP5 confocal laser scanning microscope (Leica Microsystems, Mannheim, Germany) as previously described [1, 2]. Quantification of spreading area and shape factor was performed using ImageJ (National Institutes of Health, Bethesda, Maryland, USA). To quantify attachment, cell layers were rinsed three times with PBS after an attachment period of 30 minutes, before plates were frozen at -80°C. Quantification of attached cells was performed by measuring the DNA content in each well using a Quant-iT™ PicoGreen assay (Life Technologies) according to the manufacturer's instructions. Three independent experiments were performed with at least three replicates for each condition.

Proliferation assays

BC cells were seeded at a density of 2500 cells/cm^2 on TCP or hOBM. Cell proliferation was assessed by an Alamar Blue (Life Technologies) assay. At each time point, BC cells were incubated for 2 hours in culture medium containing 8% (v/v) Alamar Blue reagent. Alamar Blue added to cell-free wells served as a background controls. Supernatants were then transferred to a 96-well black plate (Corning Incorporated, Corning, NY, USA) and fluorescence signals (excitation 544 nm, emission 590 nm) were detected using a POLARstar OPTIMA plate reader (BMG LABTECH, Offenburg, Germany). Three independent experiments were performed with at least six replicates for each condition.

BC cell proliferation and colony formation in 3D hydrogels

BC cells were embedded in 5% (w/v) gelatin methacrylate (gelMA) at a density of 7000 cells per 50 µl and gel crosslinking was performed under ultra-violet light exposure for 10 minutes, as previously described [3]. Cell proliferation was assessed over time by an Alamar Blue assay (Life Technologies). Three independent experiments were performed with at least 6 samples for each condition. After 2 weeks, cell-loaded hydrogels were fixed in 4% paraformaldehyde for 15 minutes, stained with rhodamine-conjugated phalloidin and 4',6-diamidino-2-phenylindole (DAPI) and imaged using a Leica TCS SP5 confocal laser scanning microscope (Leica Microsystems, Mannheim, Germany) as previously described [3]. BC colony size was quantified using ImageJ.

Migration assays

BC cells were seeded at a density of 15000 cells/ cm^2 on hOBM grown in 6-well plates and imaged with a Leica AF6000 LX wide-field fluorescence microscope (Leica Microsystems, Wetzlar, Germany) at 37°C and 5% CO_2. Brightfield and fluorescence images were recorded in sequence every 16 minutes for a total period of 24 hours. Image stacks were analyzed in Image J as described previously [1] and the effective distance (start-to-end distance), total distance, instantaneous speed (average frame-to-frame speed) and directionality (effective

distance/total distance) of cell migration were calculated. A minimum of 96 individual cells from a total of 12 different movies were analyzed for each condition.

qRT-PCR

For qRT-PCR analysis of tumor specimens, tissue homogenization was performed using 2 mm glass beads (Sigma-Aldrich) in a Mini-BeadBeater (Biospec Products), followed by RNA extraction using Trizol (Life Technologies) according to the manufacturer's instructions. RNA concentrations were measured with a Nanodrop ND-1000 UV-Vis Spectrophotometer (Thermo Scientific, Waltham, Massachusetts, USA) and only samples with an optical density 260/280 ratio greater than 1.6 were used. Reverse transcription of 500 ng RNA was performed using a DyNAmo cDNA Synthesis Kit (Thermo Scientific) according to the manufacturer's instructions. Primers were purchased from GeneWorks (Thebarton, South Australia, Australia). *GAPDH* and *β-actin* were used as house-keeping genes. The primer sequences are detailed in Supplementary Table 2. qRT-PCR was performed on a 7900HT Fast Real-Time PCR System (Life Technologies) using a 384-well block module, as previously described [1]. Relative gene expression was calculated as $2^\wedge(ct_{\text{geometric mean of housekeeping genes}} - ct_{\text{gene of interest}})$.

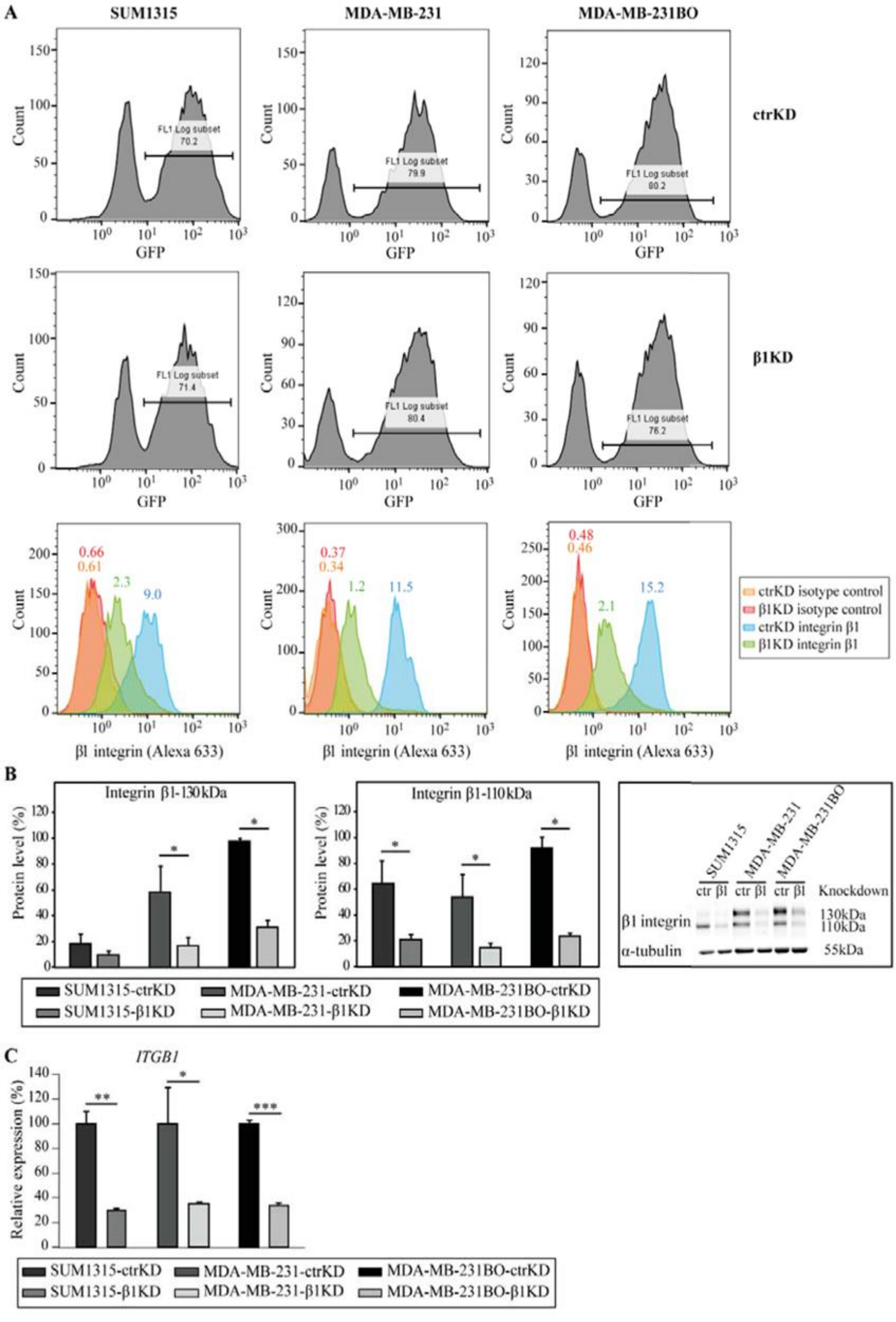

A
SUM1315
MDA-MB-231
MDA-MB-231BO
ctrKD
β1KD
Count
GFP
FL1 Log subset
70.2
79.9
80.2
71.4
80.4
76.2
β1 integrin (Alexa 633)
0.66
0.61
2.3
9.0
0.37
0.34
1.2
11.5
0.48
0.46
2.1
15.2
ctrKD isotype control
β1KD isotype control
ctrKD integrin β1
β1KD integrin β1
B
Integrin β1-130kDa
Integrin β1-110kDa
Protein level (%)
SUM1315-ctrKD
SUM1315-β1KD
MDA-MB-231-ctrKD
MDA-MB-231-β1KD
MDA-MB-231BO-ctrKD
MDA-MB-231BO-β1KD
SUM1315
MDA-MB-231
MDA-MB-231BO
ctr β1 ctr β1 ctr β1
Knockdown
β1 integrin
α-tubulin
130kDa
110kDa
55kDa
C
ITGB1
Relative expression (%)
SUM1315-ctrKD
SUM1315-β1KD
MDA-MB-231-ctrKD
MDA-MB-231-β1KD
MDA-MB-231BO-ctrKD
MDA-MB-231BO-β1KD

Supplementary Figure 1: Characterization of β1 integrin knock-down in BC cell lines SUM1315, MDA-MB-231 and MDA-MB-231BO. A: Flow cytometry analysis of GFP-positive fractions and β1 integrin levels in ctrKD and β1KD BC cell lines. B: Western blot analysis of β1 integrin protein knockdown. C: qRT-PCR analysis of β1 integrin gene expression. Data are represented as mean ± standard error.

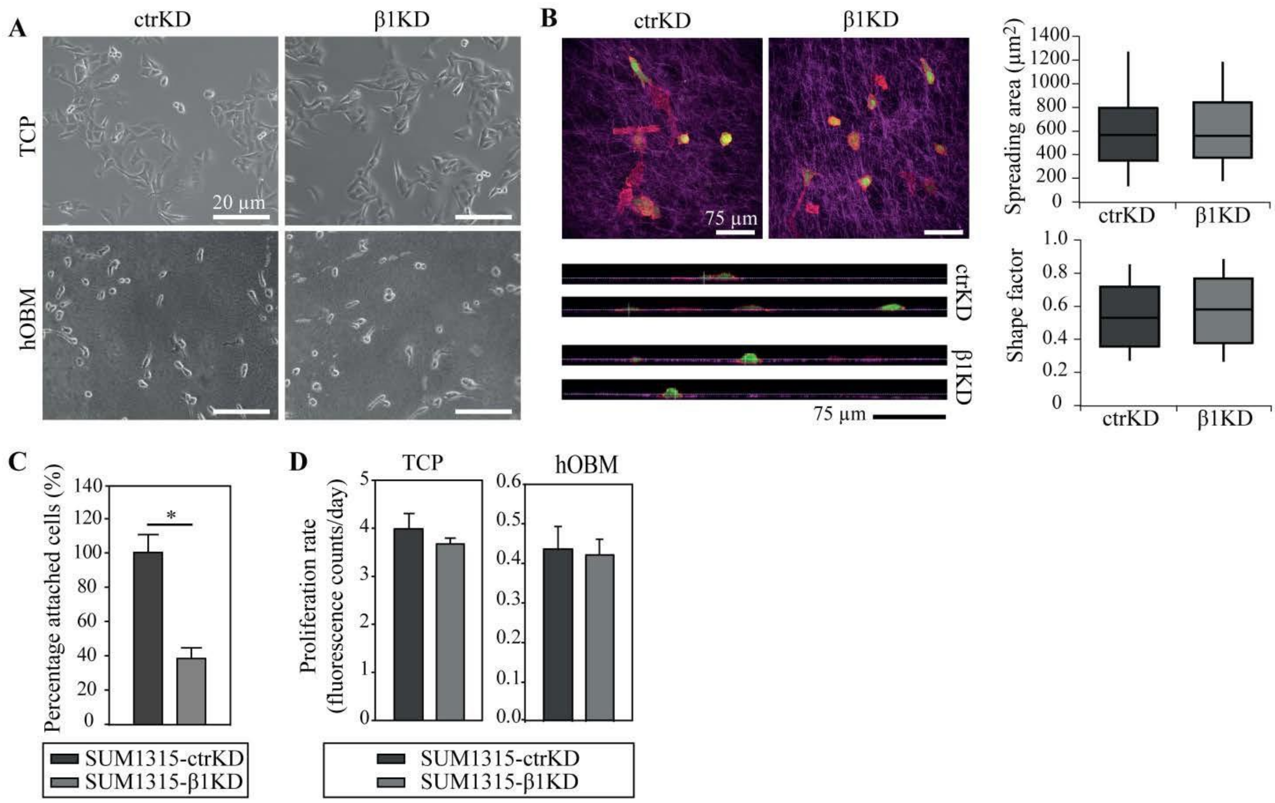

Supplementary Figure 2: β1 integrins mediate attachment but not spreading and proliferation of SUM1315 cells within hOBM. A: Representative phase contrast micrographs showing the morphology of ctrKD and β1KD SUM1315 cells grown on TCP and hOBM. B: Representative confocal z-stacks (maximal projections and orthogonal views) showing the morphology of GFP-positive (green) ctrKD and β1KD SUM1315 cells stained for F-actin (red) and nuclei (blue) on the hOBM, which is visualized by an immunofluorescence staining against human-specific fibronectin (pink). Box-plots represent the quantitative analysis of cell spreading area and shape factor. Horizontal lines show the

medians, 75th and 25th percentiles; upper and lower whiskers indicate the 90th and 10th percentiles. C: Quantification of cell attachment to hOBM. DNA content is measured after a 30 min attachment period and washing the cell layers. D: SUM1315 proliferation rates on TCP and hOBM evaluated by an Alamar Blue assay. Data are represented as mean ± standard error.

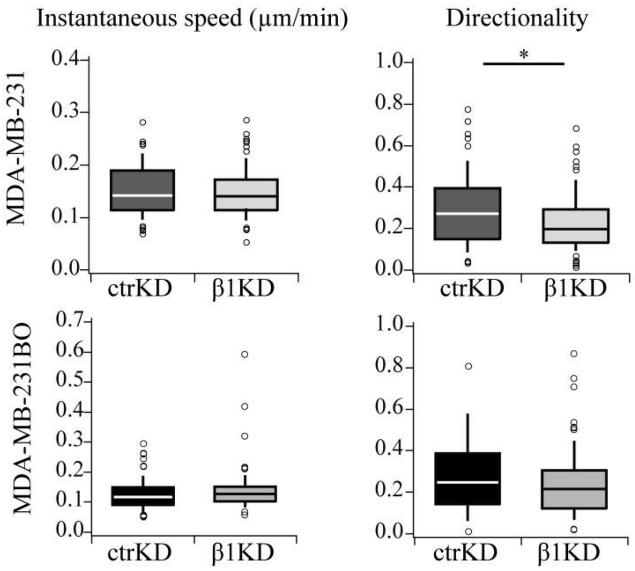

Supplementary Figure 3: β1 integrins do not influence BC cell migration on intact mineralized cell sheets. Quantification of instantaneous migration speed and directionality. Box-plots show the medians, 75th and 25th percentiles; upper and lower whiskers indicate the 90th and 10th percentiles; circles denote outliers.

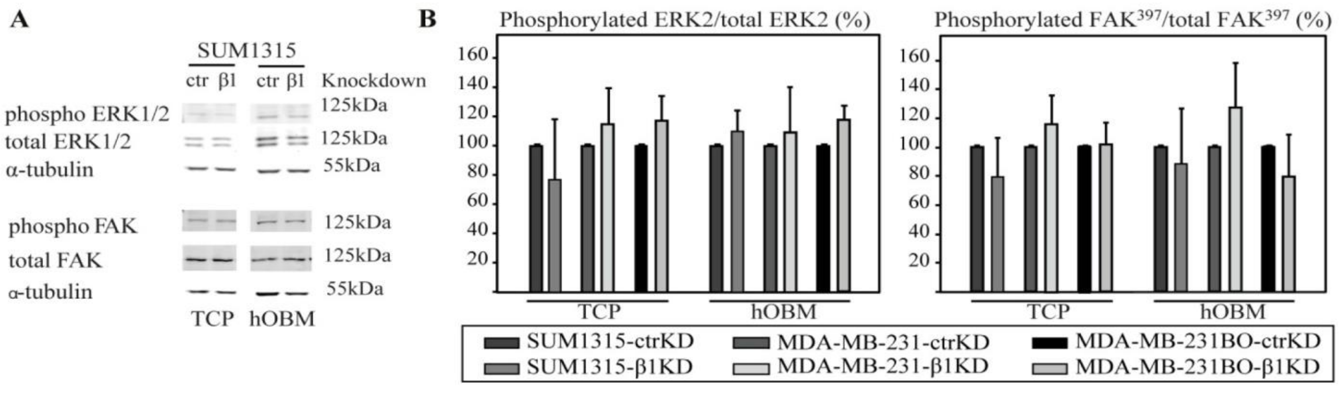

Supplementary Figure 4: Western blot analysis of ERK1/2 and FAK phosphorylation in BC cells cultured on TCP and hOBM. A: Western blots are shown for SUM1315 cells. B: Quantification of ERK2 and FAK phosphorylation in BC cells. Data are represented as mean ± standard error.

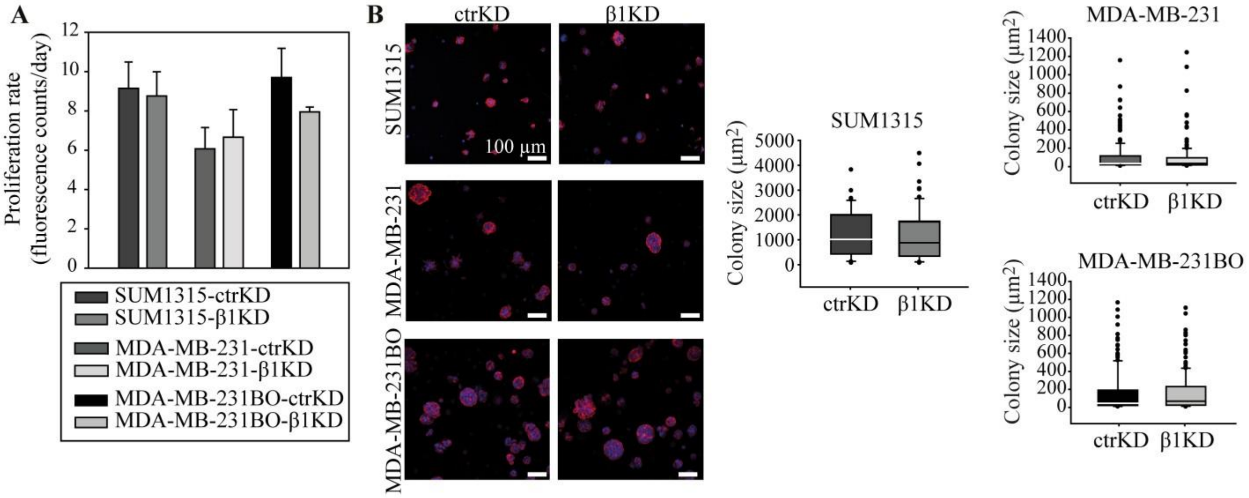

Supplementary Figure 5: β1 integrin knock-down does not affect the growth of BC cell colonies in 3D GelMA hydrogels. A: Quantification of BC cell proliferation in GelMA measured by an Alamar Blue assay. Data are represented as mean ± standard error. B: Representative confocal z-stacks showing the morphology of BC colonies stained for F-actin (red) and nuclei (blue). Quantification of colony size. Box-plots show the medians, 75th and 25th percentiles; upper and lower whiskers indicate the 90th and 10th percentiles; circles denote outliers.

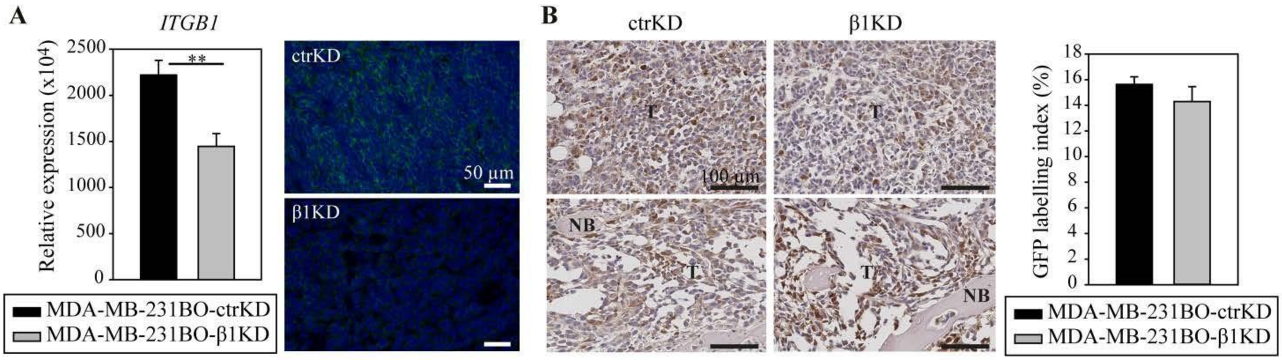

Supplementary Figure 6: β1 integrin knock-down in BC cells is stable after *in vivo* injection. A: qRT-PCR analysis indicates β1 integrin gene expression levels remain decreased in β1KD tumors compared to ctrKD. Representative IHC stainings showing β1 integrin expression (green), with nuclei counterstained (blue). B: Quantification of IHC analysis shows that GFP levels are similar between groups. Data are represented as mean ± standard error. NB: new bone, T: tumor.

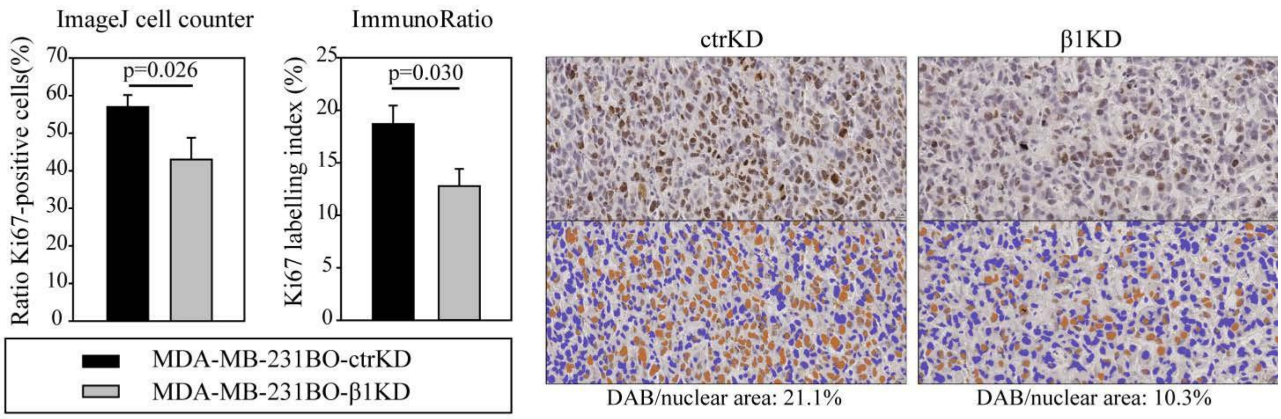

Supplementary Figure 7: Comparison of different methods of image analysis to quantify Ki67-positive cells in IHC stains. Quantification was performed using the ImageJ cell counter plug-in or the ImmunoRatio application. For the ImmunoRatio analysis representative images from the IHC stains and the corresponding pseudo-colored images with the segmented staining components are shown. Data are represented as mean ± standard error.

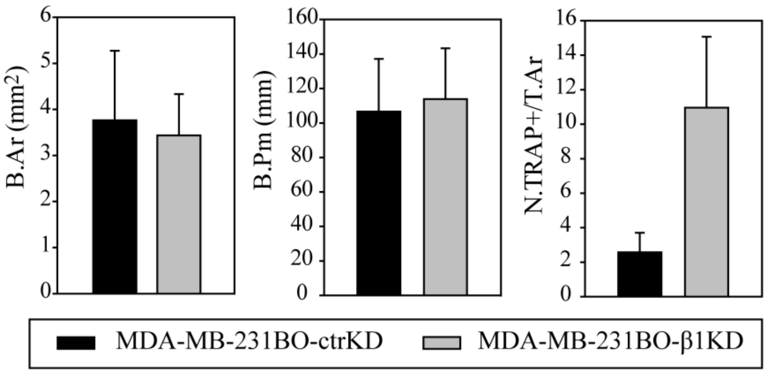

Supplementary Figure 8: Histomorphometric analyses on sections of β1KD and ctrKD cell-injected hTEBCs stained for TRAP. Quantification of mineralized tissue area (B.Ar), perimeter (B.Pm) and the number of TRAP-positive cells not adjacent to bone (not counted as osteoclasts) normalized to the total tissue area (N.TRAP+/T. Ar). Data are represented as mean ± standard error.

Supplementary Table 1: Primary antibodies and protocols used for IHC analysis

**Immunogen**	**Species reactivity**	**Source**	**Catalogue number**	**Host species**	**Dilution**	**Antigen retrieval**
Ki67	Human	Dako (Glostrup, Denmark)	M7240	mouse monoclonal	1:75	Tris-EDTA Buffer (pH 9) 95°C, 10
vWF	human, mouse cross-reactivity	Dako (Glostrup, Denmark)	IR527	rabbit polyclonal	ready to use	citrate buffer (pH 6) 95°C, 4 min
GFP	broad	Abcam (Cambridge, UK)	ab290	rabbit polyclonal	1:2000	citrate buffer (pH 6) 95°C, 4 min
NuMA	human	Epitomics (Burlingame, CA,	S2825	rabbit polyclonal	1:100	citrate buffer (pH 6) 95°C, 4 min
Integrin β1	human	Abcam (Cambridge, UK)	ab134179	rabbit monoclonal	1:100	citrate buffer (pH 6) 95°C, 20 min

Supplementary Table 2: Primer sequences for qRT-PCR analysis

**Gene**		**Primer sequences**
PTHrP	Forward:	AGCCCACCAGAGGAGGTC
	Reverse:	CTGAGACCCTCCACCGAGC
OPG	Forward:	GCGCTCGTGTTTCTGGACA
	Reverse:	AGTATAGACACTCGTCACTGGTG
IL6	Forward:	TCAGCCCTGAGAAAGGAGACATG
	Reverse:	CCAGGCAAGTCTCCTCATTGAATCC
RANKL	Forward:	GCCCCTTCGCAGCTGAAGTG
	Reverse:	GGTACAATTTGCGGCACTTGTG
ITGB1	Forward:	GCCTGTTTACAAGGAGCTGAA
	Reverse:	CTGACAATTTGCCGTTTTCC

GAPDH	Forward:	TCAGCAATGCCTCCTGCAC
	Reverse:	TCTGGGTGGCAGTGATGGC
β-actin	Forward:	TTCGAGCAAGAGATGGCCAC
	Reverse:	ACAGGACTCCATGCCCAG

References

1. Taubenberger AV, Quent VM, Thibaudeau L, Clements JA and Hutmacher DW. Delineating breast cancer cell interactions with engineered bone microenvironments. J Bone Miner Res. 2013; 28(6):1399-1411.

2. Reichert JC, Quent VMC, Burke LJ, Stansfield SH, Clements JA and Hutmacher DW. Mineralized human primary osteoblast matrices as a model system to analyse interactions of prostate cancer cells with the bone microenvironment. Biomaterials. 2010; 31(31):7928-7936.

3. Kaemmerer E, Melchels FP, Holzapfel BM, Meckel T, Hutmacher DW and Loessner D. Gelatine methacrylamide-based hydrogels: An alternative three-dimensional cancer cell culture system. Acta Biomater. 2014; 28(14):00087-00087.
